# Transcriptomic markers in pediatric septic shock prognosis: an integrative analysis of gene expression profiles

**DOI:** 10.1590/1414-431X202010152

**Published:** 2021-01-25

**Authors:** Qian Wang, Jie Huang, Xia Chen, Jian Wang, Fang Fang

**Affiliations:** 1 Anesthesiology Department, Children's Hospital of Soochow University, Suzhou, China; 2 Department of Cardiology, Children's Hospital of Soochow University, Suzhou, China; 3 Institute of Pediatric Research, Children's Hospital of Soochow University, Suzhou, China

**Keywords:** Pediatric, Septic shock, Transcriptomic markers, Co-expression network, Prognosis

## Abstract

The goal of this study was to identify potential transcriptomic markers in pediatric septic shock prognosis by an integrative analysis of multiple public microarray datasets. Using the R software and bioconductor packages, we performed a statistical analysis to identify differentially expressed (DE) genes in pediatric septic shock non-survivors, and further performed functional interpretation (enrichment analysis and co-expression network construction) and classification quality evaluation of the DE genes identified. Four microarray datasets (3 training datasets and 1 testing dataset, 252 pediatric patients with septic shock in total) were collected for the integrative analysis. A total of 32 DE genes (18 upregulated genes; 14 downregulated genes) were identified in pediatric septic shock non-survivors. Enrichment analysis revealed that those DE genes were strongly associated with acute inflammatory response to antigenic stimulus, response to yeast, and defense response to bacterium. A support vector machine classifier (non-survivors *vs* survivors) was also trained based on DE genes. In conclusion, the DE genes identified in this study are suggested as candidate transcriptomic markers for pediatric septic shock prognosis and provide novel insights into the progression of pediatric septic shock.

## Introduction

Sepsis is the world's leading cause of death of children, and it represents a complex disease with dysregulated inflammatory responses and a high mortality rate (1). An immune response initiated through an invading pathogen cannot be controlled to restore homeostasis in sepsis, which generates a pathological syndrome with sustained excessive inflammation as well as immune suppression (2). Toll-like receptors, nuclear factor κB, and cytokines including tumor necrosis factor α, interleukins 1, 2, 6, 8, and neutrophils all take part in the generation of sepsis (3). Although there are common points between adult and pediatric sepsis, crucial differences do exist in pathophysiology, clinical presentation, as well as therapeutic approaches (4).

High-throughput transcriptomic data grow rapidly, enabling gene expression profiling and identification of prognostic targets in disease. Over the last decade, several studies have explored the prognosis of pediatric septic shock by transcriptional profiling using microarrays (5–8). Integrative analysis based on multiple transcriptomic datasets could help to discover robust prognostic candidates. Therefore, in this study, the gene expression patterns of pediatric septic shock patients (of survivors and non-survivors) were investigated using public microarray datasets. The differently expressed (DE) genes identified were then further interpreted by enrichment analysis, construction of co-expression networks, and receiver operating characteristic (ROC) curve analysis.

In this study, data pre-processing, identification of DE genes, ROC analysis, and support vector machine (SVM) model training were performed using R software (http://www.r-project.org/) and bioconductor packages (9). Enrichment analysis and construction of co-expression networks were also performed using Database for Annotation, Visualization and Integrated Discovery (DAVID) (10,11) and Cytoscape (12) software, respectively.

## Material and Methods

### Microarray datasets search and selection

In this study, public microarray datasets were searched from the earliest dataset until Dec 3, 2018 using the keywords “sepsis”/“septic shock” in the Gene Expression Omnibus (GEO) database (http://www.ncbi.nlm.nih.gov/geo/) (13). Further selection of the datasets was performed for subsequent analyses. The selection criteria were: i) dataset using whole blood for gene expression analysis; ii) dataset with detailed gene expression data for both survivors and non-survivors; and iii) dataset with sample size not fewer than 30. Animal studies and adult studies were excluded.

Data collection from each eligible dataset was performed by two investigators independently. The data consisted of the sample size, GEO accession, sample source, raw gene expression data, and platform used. Final data collection was determined by checking between the two investigators.

### Data analysis methods

Four Affymetrix datasets were included in this study (3 datasets for training and 1 dataset for testing). Raw data (CEL files) of the 3 training datasets were downloaded from the GEO database, merged, and pre-processed using the R package “affy”, based on the Robust Multichip Average (RMA) method (14). Hybridization probes were then mapped to genes (Entrez IDs) based on the platform table. Probes not mapping to genes and probes mapping to multiple genes were excluded. If two or more probes mapped to the same gene, we calculated the arithmetic mean of the probe values to represent the gene expression. Using the R package “limma” (15,16), DE genes in septic shock non-survivors compared with survivors were identified based on the following criteria: i) absolute log2 fold change (LFC) greater than 0.8; and ii) false discovery rate (FDR)-adjusted P-value less than 0.05.

We further performed functional interpretation (both gene ontology (GO) analysis and Kyoto Encyclopedia of Genes and Genomes (KEGG) pathway analysis) of the DE genes identified by DAVID 6.8 (10,11). The P-value threshold was set at 0.05 in the GO analysis (17). Hypergeometric testing (P-value threshold: 0.05) was used in the pathway analysis (18). Then, calculation of Pearson's correlation coefficients was performed between the DE genes based on their expression levels. A correlation threshold of 0.8 was used, and highly correlated gene pairs were chosen to construct the co-expression network using Cytoscape software, version 3.4.0 (12). Furthermore, using the R package “pROC” (19), we evaluated the classification performance of each DE gene by ROC curve plotting and calculation of area under the ROC curve (AUC) values. Then, we performed recursive feature selection of DE genes using the R package “caret” (20) and trained an SVM model for pediatric septic shock prognosis according to the selected features using the R package “kernlab” (21) (Gaussian RBF kernel; 10-fold cross-validation). External model validation was also performed using the testing dataset.

## Results

### DE genes in pediatric septic shock non-survivors

A total of 8 studies were obtained through the original search, among which 4 adult studies were excluded. The remaining 4 microarray datasets (3 training datasets and 1 testing dataset all from the Affymetrix microarrays) (5–8) were used for subsequent analysis. Table 1 presents detailed information about the 4 datasets.

**Table 1:** Microarray datasets used in this analysis.

Dataset	GEO accession	Sample size		Sample source	Platform	Dataset usage	Submission date
		Non-survivor	Survivor				
1	GSE9692 (5)	6	24	Whole blood	Affymetrix Human Genome U133 Plus 2.0 Array	Training	2007
2	GSE26378 (6)	12	70	Whole blood	Affymetrix Human Genome U133 Plus 2.0 Array	Training	2010
3	GSE26440 (7)	17	81	Whole blood	Affymetrix Human Genome U133 Plus 2.0 Array	Training	2011
4	GSE4607 (8)	9	33	Whole blood	Affymetrix Human Genome U133 Plus 2.0 Array	Testing	2006

GEO: Gene Expression Omnibus.

**Figure 1:**
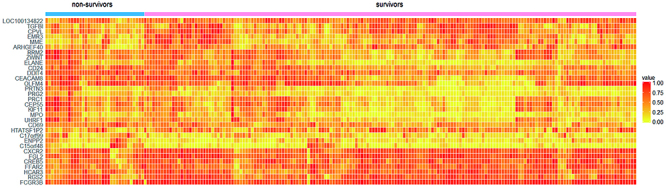
Heatmap of expression data for 32 differentially expressed genes in the collection of 3 training datasets.

For the 3 training datasets, pre-processing resulted in expression data of 20,464 genes in 35 pediatric septic shock non-survivors and 175 survivors. A total of 32 genes were found to be differentially expressed between non-survivors and survivors across the microarray datasets based on the criteria (absolute LFC >0.8, FDR-adjusted P-value <0.05) (Supplementary Table S1). Figure 1 shows the heat map of expression data for the 32 DE genes in the collection of the 3 training datasets. Among the 32 DE genes, 18 genes were up-regulated and 14 genes were downregulated. Table 2 presents the top 10 most significantly upregulated and top 10 most significantly downregulated genes. The top upregulated gene was olfactomedin 4 (OLFM4) (LFC=1.72). OLFM4 is a glycoprotein that functions in innate immunity, inflammation, and cancer (22). It was suggested to be a neutrophil subset marker in patients suffering from septic shock (23). The most downregulated gene was membrane metalloendopeptidase (MME) (LFC=-1.11). MME is widely present in organs and tissues, contributing to the inactivation of many biological peptides (24,25). Decreased MME expression capacity was detected in the neutrophils from patients with septic shock (25).

**Table 2:** The 10 most significantly upregulated and most significantly downregulated genes in pediatric septic shock non-survivors.

Entrez ID	Gene symbol	Gene full name	Log2 fold-change	False discovery rate-adjusted P-value
Significantly upregulated genes				
10562	OLFM4	olfactomedin 4	1.717146732	0.029147425
1088	CEACAM8	carcinoembryonic antigen-related cell adhesion molecule 8	1.358191894	0.035863019
84419	C15orf48	chromosome 15 open reading frame 48	1.283283163	0.002649534
1991	ELANE	elastase, neutrophil-expressed	1.162470483	0.022391364
5657	PRTN3	proteinase 3	1.104928095	0.005347329
54541	DDIT4	DNA damage inducible transcript 4	1.069842902	0.000011715
5553	PRG2	proteoglycan 2, pro eosinophil major basic protein	1.01469606	0.000011715
100133941	CD24	CD24 molecule	1.014691224	0.001427259
6241	RRM2	ribonucleotide reductase regulatory subunit M2	1.001889643	0.014703381
55165	CEP55	centrosomal protein 55	1.00180377	0.001823254
Significantly downregulated genes				
4311	MME	membrane metalloendopeptidase	-1.111678792	0.005267437
10875	FGL2	fibrinogen-like 2	-1.099276289	0.000425565
8843	HCAR3	hydroxycarboxylic acid receptor 3	-1.083432919	0.027926014
100134822	LOC100134822	LOC100134822	-1.065641306	0.003118448
3579	CXCR2	C-X-C motif chemokine receptor 2	-1.028150049	0.001756143
5997	RGS2	regulator of G protein signalling 2	-0.962605787	0.008475327
2215	FCGR3B	Fc fragment of IgG receptor IIIb	-0.923733902	0.001756143
54504	CPVL	carboxypeptidase, vitellogenic-like	-0.922107067	0.017614822
2867	FFAR2	free fatty acid receptor 2	-0.84291548	0.015277801
401233	HTATSF1P2	HIV-1 Tat specific factor 1 pseudogene 2	-0.837903188	0.022307329

### Enrichment analysis and co-expression network construction

**Figure 2:**
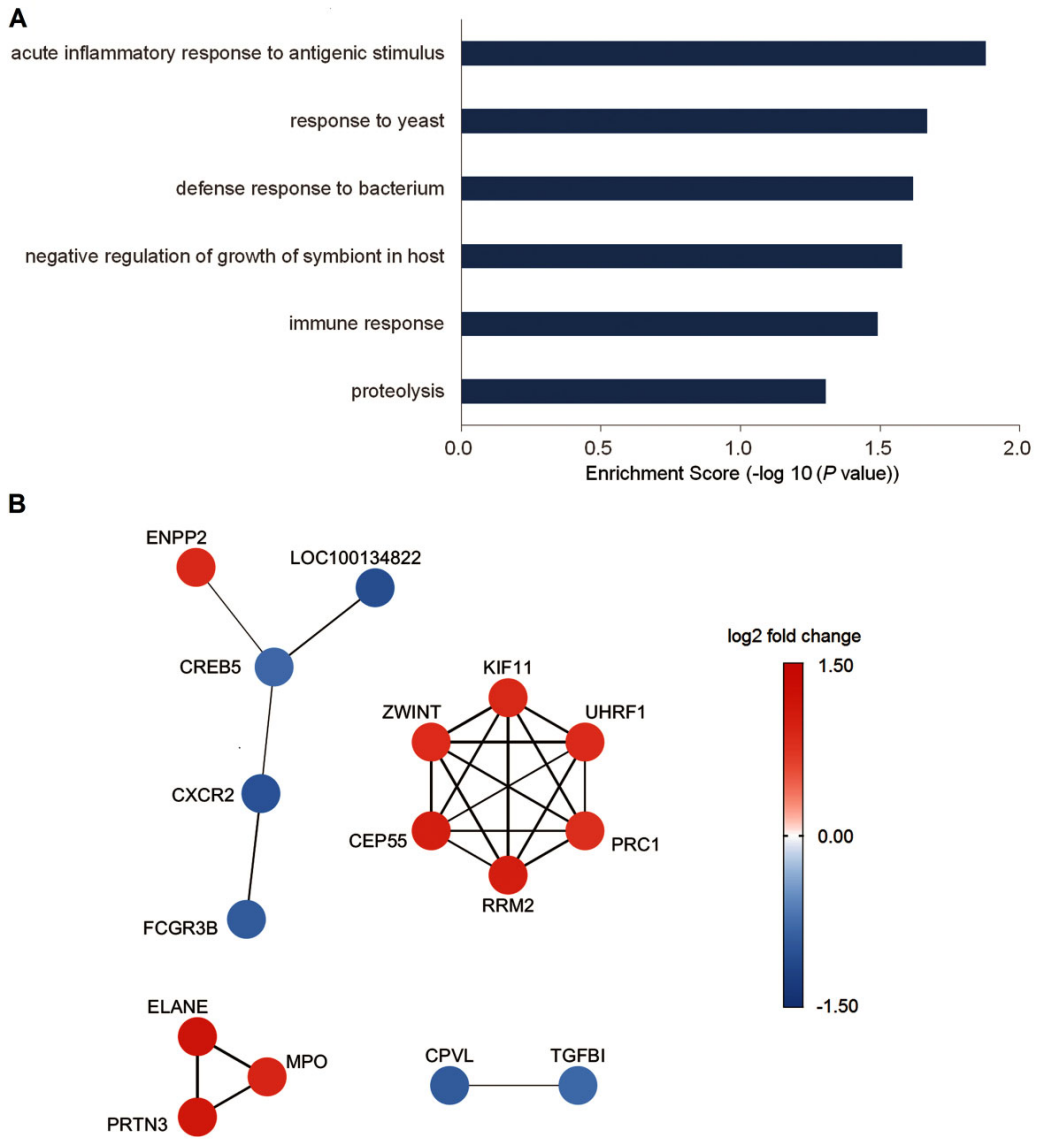
Enrichment analysis and co-expression network construction results. **A**, Summary of the gene ontology analysis results for the differentially expressed (DE) genes in pediatric septic shock non-survivors. **B**, Co-expression network of highly correlated DE gene pairs in pediatric septic shock non-survivors (correlation coefficients >0.8). Edge widths are proportional to the correlation coefficients.

For further functional investigation, we performed advanced analyses (GO and pathway analysis) of the DE genes. Figure 2A and Supplementary Table S2 show the results of GO biological process analysis, which were similar to those reported in the original microarray studies (5–8). The DE genes were significantly enriched in 6 GO terms, and the top 3 were “acute inflammatory response to antigenic stimulus” (P=0.013), “response to yeast” (P=0.021), and “defense response to bacterium” (P=0.024). In the pathway analysis, when DE genes were mapped to the KEGG database, no significant pathway was identified.

We also constructed a co-expression network using significantly correlated DE gene pairs (correlation coefficients greater than 0.8). The co-expression network contained 16 nodes and 23 edges (Figure 2B). We further extracted sub-networks for the GO enrichment analysis. One GO term (“chemotaxis”) was significantly enriched in the sub-network composed of ENPP2, LOC100134822, CREB5, CXCR2, and FCGR3B. In the sub-network containing ELANE, MPO, and PRTN34, 4 significant GO terms (“response to yeast”, “negative regulation of growth of symbiont in host”, “defense response to bacterium”, and “response to lipopolysaccharide”) were identified.

### Classification performance evaluation and validation

**Figure 3:**
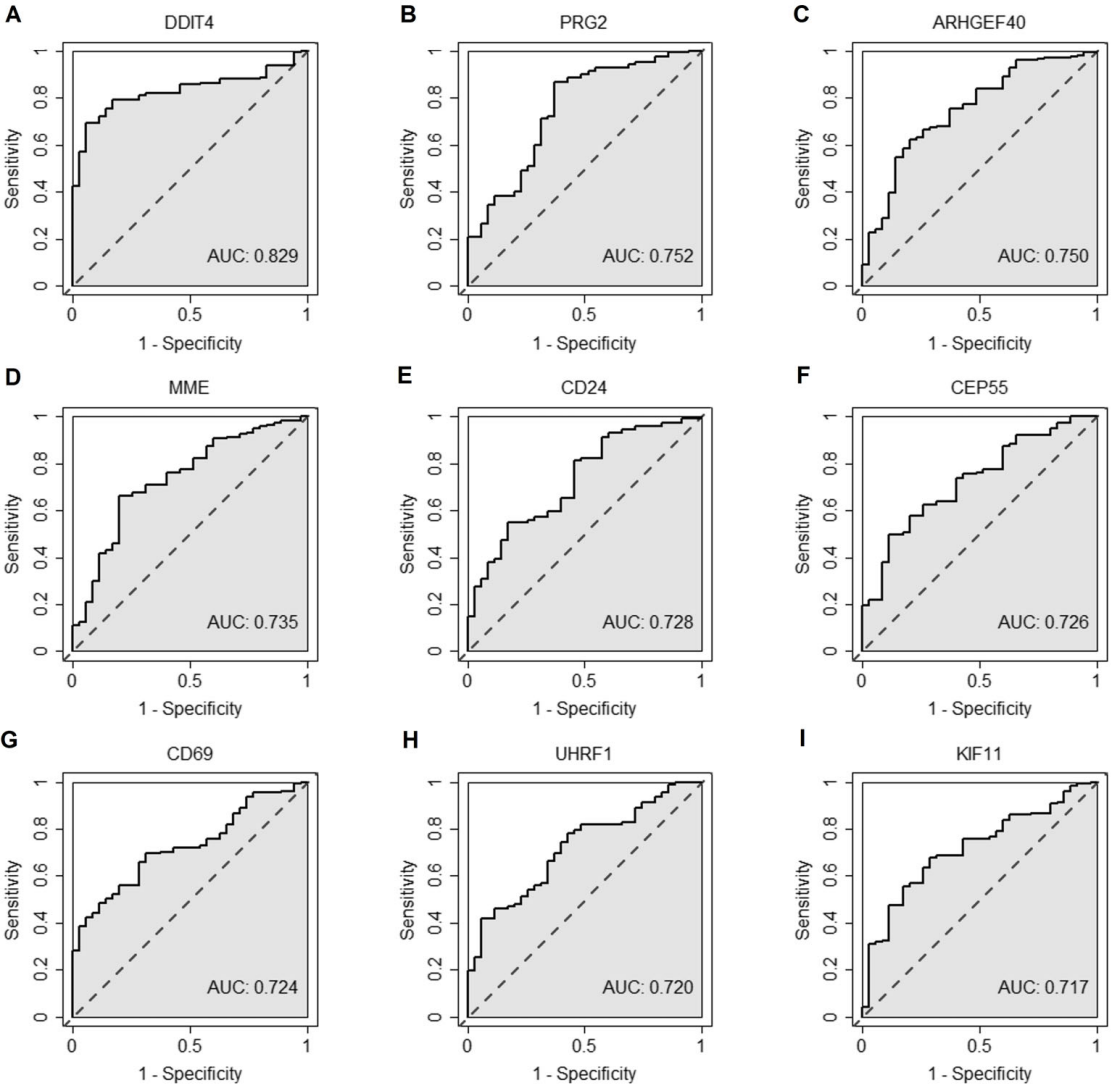
Receiver operating characteristic curves of top performing differentially expressed genes in prognostic prediction of pediatric septic shock. **A**, DDIT4; **B**, PRG2; **C**, ARHGEF40; **D**, MME; **E**, CD24; **F**, CEP55; **G**, CD69; **H**, UHRF1; **I**, KIF11. AUC: area under the receiver operating characteristic curve.

**Figure 4:**
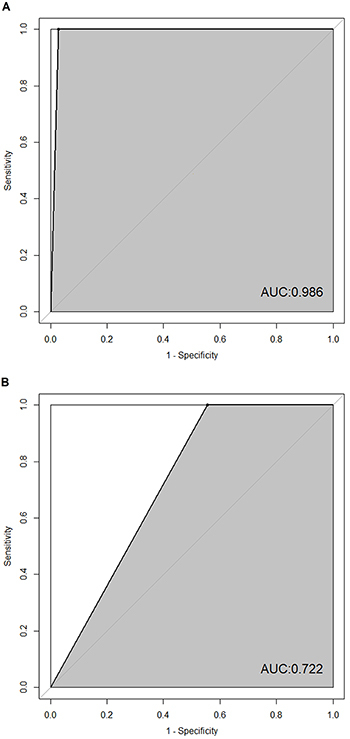
Receiver operating characteristic curves of the support vector machine classifier in prognostic prediction of pediatric septic shock. **A**, Performance with the training dataset. **B**, Performance with the testing dataset. AUC: area under the receiver operating characteristic curve.

ROC curves and AUC values suggested that the top performing DE genes were DDIT4, PRG2, ARHGEF40, MME, CD24, CEP55, CD69, UHRF1, and KIF11 (Figure 3), with the best single gene's AUC as great as 0.829. The results of recursive feature selection showed that 11 genes (Supplementary Table S3) were sufficient to achieve close to 91% prediction accuracy. Based on the selected features, we trained an SVM classifier (pediatric septic shock non-survivors *vs* survivors) and evaluated its performance by 10-fold cross-validation (cross-validation error: 0.133). The SVM classifier performed well in the training dataset (AUC=0.986, Figure 4A). The pre-processed testing dataset was also used for independent validation, and the SVM classifier was constructed according to the training datasets, showing modest performance in the testing dataset (AUC=0.722, Figure 4B).

## Discussion

Many genes are reported to be associated with septic shock mortality (23,26,27). It is a challenge currently to identify the most important candidate genes and pathways in septic shock prognosis. Growth of high-throughput transcriptomic data has enabled the integrative analysis of multiple datasets to discover robust candidates for prognosis and treatment. Therefore, to identify potential transcriptomic markers in pediatric septic shock prognosis, we performed an integrative analysis of multiple public microarray datasets.

In this study, 32 DE genes were identified in pediatric septic shock non-survivors, among which 18 genes were upregulated and 14 were downregulated. The most upregulated gene was OLFM4. The most downregulated gene was MME. Among the 32 DE genes identified, OLFM4 was reported to be present in only 20-25% of peripheral blood neutrophils and identified a subset of human neutrophils (28). MME is widely present in organs and tissues, contributing to the inactivation of many biological peptides (24,25). Decreased MME expression capacity was detected in the neutrophils from patients with septic shock (25). CXCR2 is a key stimulant to immune cell migration as well as recruitment, especially for neutrophils. The CXCR2 signaling pathway is suggested to be a potential target for modification of neutrophil dynamics in inflammatory disorders (29). CEACAM8 belongs to CEA family, which is known to function as intercellular adhesion molecules. CEACAM8 is suggested to be involved in the regulation of the neutrophil adhesion (30). ELANE encodes neutrophil elastase, which is a cytotoxic serine protease stored in azurophil granules and released after neutrophil activation. ELANE also functions in neutrophil extracellular traps (31). Neutrophil dysfunction is known to promote sepsis, and the alterations of chemokines, cytokines, as well as other mediators lead to neutrophil dysfunction in sepsis. Thus, the DE genes identified in this study, such as OLFM4, MME, CXCR2, CEACAM8, and ELANE, probably take part in pediatric sepsis development by regulation of neutrophil function (32).

Furthermore, a series of DE genes identified in this study have not yet been investigated in pediatric septic shock prognosis, and the exact contributions of these genes to pediatric septic shock are not yet clear. Since these DE genes could serve as potential transcriptomic markers for pediatric septic shock, further research is required.

In the enrichment analysis of the 32 DE genes, “response to yeast” (P=0.021) was the second most enriched GO term. This is interesting because yeast infection has been reported to cause sepsis (33).

According to the literature, machine learning methods, including SVM, usually have high prediction accuracy (34). Hence, in this study, we trained an SVM classifier (pediatric septic shock in non-survivors *vs* survivors) using gene expression data to improve the performance of prognostic prediction. The SVM classifier performed well in the training dataset with an AUC value of 0.986 (Figure 4A), compared with Pediatric Sepsis Biomarker Risk Model-II (35). However, it showed modest performance in the testing dataset (AUC=0.722, Figure 4B), suggesting that there could be an over-fitting problem. To the best of our knowledge, no gene expression-based SVM model has yet been reported for pediatric septic shock prognostic prediction. We hope the predictive model trained in this study facilitates better prognosis and treatment of pediatric septic shock.

Because of the existence of several limitations, the results of our study should be considered cautiously. First, the sample size used in our study was insufficient. Second, stratified analyses according to potential influential factors (age, sex, and platform usage) could not be performed in this study due to the lack of corresponding data. Third, both the biological knowledge base and the pathway data are currently incomplete. In consideration of the reported impact of age and sex on pediatric sepsis patients (36), stratified analyses, based on factors including age, sex, and platform usage, are required in the future. Further analysis based on larger sample sizes is also necessary. Moreover, to explore the exact roles of candidate DE genes in pediatric septic shock, experimental verification and functional studies of these DE genes must also be performed in the future.

In conclusion, consistently DE genes in pediatric septic shock non-survivors were identified in this study, and they could be potential transcriptomic markers. GO analysis suggested that these candidates were strongly correlated with acute inflammatory response to antigenic stimuli, response to yeast, and defense responses to bacteria. We also trained an SVM classifier (non-survivors *vs* survivors) using candidate transcriptomic markers. These results provided novel insights into the prognosis of pediatric septic shock and promoted the generation of prognostic gene sets.

## Supplementary Material

Click here to view [pdf].
